# Influence of Heterogamy by Religion on Risk of Marital Dissolution: A Cohort Study of 20,000 Couples

**DOI:** 10.1007/s10680-016-9398-9

**Published:** 2016-09-19

**Authors:** David M. Wright, Michael Rosato, Dermot O’Reilly

**Affiliations:** 1 Centre for Public Health, Institute of Clinical Sciences - Block B, Royal Victoria Hospital, Queen’s University Belfast, Grosvenor Road, Belfast, BT12 6BA UK; 2 Bamford Centre for Mental Health and Wellbeing, University of Ulster, Londonderry, UK

**Keywords:** Heterogamy, Marital dissolution, Religion, Segregation, Longitudinal study

## Abstract

Heterogamous marriages, in which partners have dissimilar attributes (e.g. by socio-economic status or ethnicity), are often at elevated risk of dissolution. We investigated the influences of heterogamy by religion and area of residence on risk of marital dissolution in Northern Ireland, a country with a history of conflict and residential segregation along Catholic–Protestant lines. We expected Catholic–Protestant marriages to have elevated risks of dissolution, especially in areas with high concentrations of a single religious group where opposition to intermarriage was expected to be high. We estimated risks of marital dissolution from 2001 to 2011 for 19,791 couples drawn from the Northern Ireland Longitudinal Study (a record linkage study), adjusting for a range of compositional and contextual factors using multilevel logistic regression. Dissolution risk decreased with increasing age and higher socio-economic status. Catholic–Protestant marriages were rare (5.9 % of the sample) and were at increased risk of dissolution relative to homogamous marriages. We found no association between local population composition and dissolution risk for Catholic–Protestant couples, indicating that partner and household characteristics may have a greater influence on dissolution risk than the wider community.

**Electronic supplementary material:**

The online version of this article (doi:10.1007/s10680-016-9398-9) contains supplementary material, which is available to authorized users.

## Introduction

Marital dissolution has profoundly negative impacts on both the individuals involved and society in general (Bracke et al. 2010; Prigerson et al. 1999; Sbarra et al. 2011; Seltzer 1994). Whilst rates vary widely among countries, many predictors of dissolution are similar (Amato and James 2010; Andersson 2003). Partner characteristics that increase the risk of marital dissolution include low income (Jalovaara 2013), unemployment (Hansen 2005), young age at marriage (Bumpass and Sweet 1972), previous cohabitation (Berrington and Diamond 1999) and a family history of divorce (Amato and DeBoer 2001; Bumpass et al. 1991). Urban residence has also been associated with increased dissolution risk (Kulu 2012; Frimmel et al. 2012) perhaps due to a greater availability of alternative partners in urban marriage markets (South and Lloyd 1995). Dissolution risk also varies with marital duration, increasing initially before stabilising, the latter often coincident with the birth of children or accumulation of significant wealth (Chan and Halpin 2003; Kulu 2014; Weiss and Willis 1997).

A major predictor of decreased marital stability is partner dissimilarity; US longitudinal studies indicate that marriages that cross age, social, religious or educational boundaries (heterogamous marriages) are at greater risk of dissolution than similarly defined homogamous marriages (Bumpass and Sweet 1972; Tzeng 1992; Heaton 2002). Stronger heterogamy effects (greater differentials in dissolution risk between heterogamous and homogamous couples) have been observed for couples crossing ethnic or native/immigrant boundaries (Dribe and Lundh 2012; Frimmel et al. 2012; Kalmijn et al. 2005; Milewski and Kulu 2014; Bratter and King 2008) although a recent US study found that homogamous marriages among Blacks had higher dissolution risk than interracial marriages (Jones 2010). Dissolution risks may also vary according to the distribution of characteristics between sexes (Bumpass et al. 1991; Call and Heaton 1997; Dribe and Lundh 2012; Vaaler et al. 2009). For example, couples in Great Britain in which the man was 15 years or more the senior were at much lower risk of dissolution than those in which the age differential was reversed (Feng et al. 2012). The increased risk of dissolution associated with many types of heterogamous marriage may stem from unresolved differences in values and attitudes (Clarkwest 2007; Sherkat 2004) or from external pressures. For example, among couples heterogamous by education in the USA, post-marital changes in labour division contrary to broad social norms have been associated with decreased marital stability (Tzeng 1992). Conversely, in cases where heterogamous marriage conforms with social norms, risk of dissolution may be reduced [e.g. couples heterogamous by earnings in which partners conform to traditional husband—breadwinner, wife—homemaker gender roles (Weisshaar 2014)].

Religious heterogamy in terms of both practice and affiliation is an established risk factor for dissolution in the USA and some European countries (Kalmijn et al. 2005; Lehrer and Chiswick 1993; Vaaler et al. 2009; Heaton 2002). A large population-based longitudinal study in the Netherlands revealed the strongest heterogamy effects (greatest increases in risk relative to homogamous couples) among marriages crossing social and historical rather than doctrinal boundaries, with Catholic–Protestant and Jewish–Gentile marriages at greater risk of dissolution than couples of mixed Protestant affiliation (Kalmijn et al. 2005). Both attitudinal differences and external pressures may increase dissolution risk for marriages crossing cultural boundaries of religion or race. Groups may have differing expectations of family life (Vaaler et al. 2009; Marks 2005), and choice of a spouse from another cultural group may violate strong social norms and carry a stigma (Lehrer and Chiswick 1993).

The relative influence of attitudinal differences and external pressures on dissolution risk of marriages heterogamous by religion has seldom been explored, so we investigated these effects using data from Northern Ireland, a country where religious practice is relatively common and where a profound divide exists between Protestants and Catholics (Lloyd and Robinson 2011; O’Malley and Walsh 2013; Brewer et al. 2013). Religious affiliation of partners was used to represent attitudinal differences, and residential segregation by religion was used as a proxy for external pressures.

Historically, the social barriers to Protestant–Catholic intermarriage were strong, with hostile views and policies held by both groups towards intermarriage. Most importantly, there have been less than two decades of relative peace following a prolonged period of violent sectarian conflict (‘the Troubles’) in which over 3600 people were killed and many more wounded (Morrissey et al. 1999). The Troubles exerted a heavy toll on Protestant–Catholic couples who were frequently forced to leave areas dominated by one community or the other and residential segregation remains one of the key manifestations of the religious divide in Northern Ireland (Boal 2002; Lloyd and Shuttleworth 2012; Shuttleworth et al. 2013; Doherty and Poole 1997).

Therefore, we expected that marriages crossing the Catholic–Protestant boundary would have elevated dissolution risk relative to the corresponding homogamous marriages. Using the relative concentrations of the main religious groups as a measure of segregation, we investigated whether dissolution risks of heterogamous Catholic–Protestant marriages were elevated in more segregated areas where approval of such marriages was expected to be lowest.

We addressed two main questions: (a) Does heterogamy by religion increase the risk of marital dissolution in Northern Ireland? and (b) Does the risk of marital dissolution for Catholic–Protestant couples increase with the degree of residential segregation by religion? We first present a further review of the evidence surrounding marital heterogamy effects, followed by background information on the social and historical context in which this study was conducted.

## Background

Hypotheses invoking both intra-couple and external social forces have been advanced to explain how partner dissimilarity can influence marital dissolution. In terms of intra-couple dissimilarity, partners with diverse characteristics and family backgrounds may have different attitudes and values regarding marriage and spousal roles (Bumpass and Sweet 1972; Bumpass et al. 1991; Tzeng 1992) and fewer opportunities for shared activities (Kalmijn et al. 2005). Differences in expectation may be most pronounced surrounding division of labour, childcare and attitudes to the extended family. This explanation has been applied in the case of African American couples in the USA, who have higher rates of dissolution than couples belonging to other ethnic groups. A longitudinal study (Clarkwest 2007) found that African American couples had greater dissimilarity in attitudes (towards sexuality, ideal family size, maternal employment and independence) and behaviours (religious attendance), measures that taken together were associated with elevated dissolution risk independent of socio-economic status. Negotiating and resolving differences in partner expectation are a normal feature of marriage, and it was suggested that unresolved differences within African American couples were due to relatively slow convergence of attitudes in the early years of marriage (Clarkwest 2007).

Increased dissolution risk for heterogamous marriages may instead be framed as a response to external social pressures if couple compositions or behaviours violate social norms. For example, analysis of the US National Longitudinal Surveys revealed that couples heterogamous by age (husband more than 3 years older than wife) or education and therefore not conforming to social norms, were up to a third more likely to divorce than couples homogamous by age or education (Tzeng 1992). Similarly, for some characteristics where heterogamy is the norm, it was associated with reduced dissolution risk; couples conforming to traditional gender roles with regard to paid employment (i.e. husband employed, wife homemaker) had reduced risk relative to couples with other employment statuses.

Empirical studies have concentrated on estimating the size of heterogamy effects rather than exploring the nature of external pressures which may include reduced family support and negative reactions from strangers (Bratter and King 2008; Kalmijn et al. 2005). There is evidence that the influence of heterogamy on marital dissolution increases with the extent of deviation from social norms. For example, dissolution risk of married couples in the UK increased with increasing age differentials between partners, especially when the woman was the older partner (Feng et al. 2012).

### Intergroup Heterogamy (Exogamy)

Heterogamy effects may also occur where marriages cross social group boundaries violating social norms favouring endogamous marriage (Dribe and Lundh 2012; Lehrer and Chiswick 1993). As with other forms of heterogamy, it might be expected that heterogamy effects would increase with increasing social distance between groups, as the distance between partners both attitudinally and in terms of external pressures would be increased (value dissimilarity hypothesis—Dribe and Lundh 2012). Here we review some examples of intergroup heterogamy and discuss the extent to which they support this hypothesis.

An investigation of over 5500 marriages in the USA found that dissolution risks were generally higher for couples heterogamous by ethnicity but that there was considerable variation in risk according to the ethnic composition of the couples (Bratter and King 2008). For example, White female/Black male couples were at twofold higher risk than homogamous White couples. Likelihood of intermarriage was used to measure intergroup distance as this metric integrates across historical intergroup relations (especially conflict between Blacks and Whites) and heterogamous couples at highest risk of dissolution were those that spanned the greatest social distances. Heterogamy effects also occur where there are social differences in group attitudes and behaviour but no history of intergroup conflict. The Swedish-speaking minority in Finland has a history of peaceful coexistence with Finnish speakers, equal constitutional status, access to Swedish-speaking educational and social institutions (Finnäs 1997) and historically higher socio-economic status (O’Leary and Finnäs 2002). However, large-scale population register-based studies have revealed that marriages crossing this ethno-linguistic divide were at marginally (9–28 %: Finnäs 1997; Saarela and Finnäs 2014b) higher dissolution risk than constituent homogamous marriages.

Intergroup heterogamy by religion has been associated with increased dissolution risk in multiple countries. In a US-based study (Lehrer and Chiswick 1993), risk of dissolution during the first 5 years was estimated for marriages classified according to religious affiliation of the spouses. Heterogamy effects increased with increasing group disparity measured in terms of social distance by belief, practice and tolerance of other religious groups. For instance, heterogamy effects for marriages heterogamous among Protestant denominations were less than for those between Protestants and Catholics. Similar findings were reported for a much larger study of heterogamy by religion and nationality in the Netherlands (Kalmijn et al. 2005). This study also found large heterogamy effects between religious groups that are separated by social boundaries rooted in history but that now hold very similar values (e.g. Catholics and conservative Reformed Protestants), indicating strong persistence of social boundaries once established. Finally, stronger heterogamy effects were observed for nationality than religion. High dissolution risk in cross-national couples may reflect the influence of negative social pressures experienced by immigrants (Dribe and Lundh 2012), but some dissolutions may have resulted from marriages of convenience between immigrants and natives to secure residency (Kalmijn et al. 2005).

In summary, in support of the value dissimilarity hypothesis, there is strong evidence that heterogamy effects increase with increasing social distance between groups defined along linguistic, ethnic, religious and nationalistic lines. In each case, not only the current but also the historical relationships between groups are important.

### The Northern Irish Context

It has been argued that the social divide in Northern Ireland is primarily ethno-national and that religion currently serves as an indicator of group identity rather than grounds for further division (O’Malley and Walsh 2013; Lloyd and Robinson 2011). There are separate school systems, a high degree of residential segregation (Lloyd and Robinson 2011) and residual differentials in socio-economic status between Protestants and Catholics. Cultural differences among religious groups are associated with variation in mortality and morbidity rates, reflecting pronounced differences in lifestyle and health behaviours (O’Reilly and Rosato 2008; O’Reilly and Rosato 2010).

The Protestant/British/Unionist community can be traced back to waves of immigration (often state sponsored) from England and Scotland that began in the sixteenth century, whereas the Catholic/Irish/Nationalist community is primarily descended from the original inhabitants of Ireland. Protestant settlers established dominancy in the north-east of Ireland, holding a privileged position with high social status by the start of the twentieth century. During the subsequent war of independence and partition of Ireland, Protestants played a key role in ensuring that Northern Ireland remained united with Great Britain. In the late 1960s, tensions over the constitutional status of Northern Ireland and civil rights for the Catholic minority erupted into violence that persisted for 30 years.

Residential segregation, already evident by the start of the Troubles, was exacerbated by each outbreak of violence with local minorities feeling threatened and sometimes forced to leave areas dominated by the other community (Lloyd and Shuttleworth 2012; Boal 2002; Doherty and Poole 1997). Following the cessation of violence, policies favouring segregated social housing were continued and so segregation remains a prominent feature of the religious divide, especially in working-class areas (Boal 2002; Lloyd and Shuttleworth 2012; Shuttleworth et al. 2013; Doherty and Poole 1997). Protestant–Catholic couples, belonging exclusively to neither community, were at high risk of displacement during the Troubles and would still be viewed with distrust in the most segregated areas.

Negative social pressures at the local-level compound institutionalised barriers to Protestant–Catholic intermarriage, for example the requirements of the Catholic Church that such marriages must be authorised by a bishop (Lloyd and Robinson 2011) and that any children must be brought up Catholic. Historically, the Catholic Church enforced a more strict prohibition of divorce than the mainstream Protestant churches (in the Catholic dominated Republic of Ireland there was a constitutional ban for several decades) but both groups retain conservative attitudes towards marital dissolution (O’Malley and Walsh 2013). However, divorce rates have increased in recent decades indicating that the influence of the Churches is declining in this area.

Estimates for the prevalence of Protestant–Catholic marriages during the Troubles are scarce because religion of partners is not recorded on marriage registration forms (Lloyd and Robinson 2011) but social attitude surveys from the last decade of the Troubles indicated a slight increase in heterogamous relationships (not necessarily marriages) from 6 % in 1989 to 9 % in 1998 (Wigfall-Williams and Robinson 2001). Protestant–Catholic marriage has been associated with worse mental but not physical health of partners in comparison with those in homogamous marriages, highlighting the detrimental effects of the pressures facing those in heterogamous marriages in contemporary Northern Ireland (McAloney 2013).

## Methods

### Data Sources

Data were drawn from the Northern Ireland Longitudinal Study (NILS), a linkage of health card registration data, the 2001 and 2011 Census returns and administrative data from other sources for a representative 28 % sample of the Northern Ireland population (O’Reilly et al. 2012). Membership of the NILS is determined by birth date; all those with one of 104 designated birth dates in the calendar year are included. Almost the entire population is registered for a health card (needed to access free health and social care). Registrations of members are matched to Census returns using a range of automated and clerical methods. From the NILS dataset, we identified a cohort of 22,900 married couples where both partners were NILS members, the religious affiliation of both partners was recorded (not imputed) and where both partners were aged from 16 to 74 at 2001.

NILS members were matched (identified) across Censuses provided that they were resident in Northern Ireland on both Census dates. Matching methods account for name changes at marriage by allowing matches to be made to both current and previous names. Census return is a legal requirement, and population response rates in 2001 and 2011 were high (95 and 92 %, respectively—NISRA 2014).

Of the marriages in our dataset, we excluded 2535 (11 %) where a death had occurred during follow-up. We also excluded 568 couples where neither member was successfully matched across Censuses. The overall matching rate was 86.4 %. Finally, to comply with NILS disclosure rules, we excluded six couples who were members of very small subgroups, leaving a total of 19,791 couples in the modelling dataset. Attrition rates by religion are given in supplementary material, Table S1.

The status of the marriages in 2011 was determined using a combination of measures. Marriages in which partners no longer shared a household in 2011 were deemed to have been dissolved. This included cases where one partner was not matched in 2011, most likely as a result of emigration. Where both partners continued to share a household in 2011, responses to the marital status Census question were used to determine whether the marriage remained intact (i.e. where both partners described themselves as married). Marriages in which either partner described themselves as no longer married (either separated or legally divorced) were deemed to have been dissolved. We were unable to control for overall marriage duration as the Census provides no measure of duration.

### Measurement of Religious Affiliation

Three linked Census questions were used to produce a composite measure of religious affiliation termed community background. The first asked whether respondents regarded themselves as belonging to a religion and directed them to one of two subsidiary questions depending on the response. If positive, respondents were then asked which religion they belonged to (i.e. their current religion). If negative, they were asked which religion they were brought up in. The majority of the sample (about 90 %) claimed a current religious affiliation and so our measure of community background reflected current religion more strongly than religion brought up in. Religion brought up in was used only if no current religion was reported. The Census did not elicit information on religious practice from respondents.

### Statistical Analysis

We fitted a series of logistic regressions to estimate the probability of marital dissolution during the 10-year follow-up period, classifying marriages by self-reported religious affiliation of the partners. Initial estimates of dissolution risk for each union type were compared with adjusted estimates using multiple linear regression for a range of variables which have previously been associated with variation in dissolution risk among couples or which are indicators of partner dissimilarity. Partner age, country of birth and three indicators of socio-economic status were included: educational attainment (university degree, no degree), economic activity (employed, inactive) and housing tenure (owner occupation, social renting, privately renting) as a proxy for accumulated wealth. Two indicators of the presence of dependent children in the household were included: those under five years and those of five or more. Also included was a three-way classification of the level of urbanisation based on settlement size [urban—the two largest cities; intermediate—towns and intermediate areas; and rural—open space and settlements of <1000 people (NISRA 2005)]. All explanatory variables were measured at the 2001 Census prior to any dissolution events, enabling straightforward interpretation of the associations with dissolution risk. For some of the variables, especially economic activity and housing tenure, model endogeneity would have been introduced if measured in 2011 as there are plausible bidirectional causal links between these variables and dissolution (e.g. living in social housing might increase marital stress and risk of dissolution, but dissolution might increase the risk of one or other partner being allocated social housing).

The associations between candidate variables and dissolution risk were estimated both singly in univariate models and in combination in multiple regressions. In both unadjusted and adjusted models, variation among areas of residence (classified into 890 Census Super Output Areas, SOAs, each with approximately 2000 residents) was modelled using random effects.

We used model comparison to test whether dissolution risk varied with the proportion of the population in each SOA with Catholic community background (a proxy for residential segregation). Taking the best-fitting multivariable model as our base, we produced three additional candidate models incorporating this measure, with functional forms representing linear, quadratic and cubic associations between proportion Catholic and dissolution risk. The relative fit of all four models was then assessed using likelihood ratio tests and AIC. Regressions were fitted using the *lme4* (Bates et al. 2014) package in *R* 3.02 (R Development Core Team 2012).

### Sensitivity Analysis

Those brought up in a particular religion but no longer declaring a current affiliation to it may have less traditional attitudes to marriage than those with a current religious affiliation, potentially leading to higher dissolution risk. We performed a sensitivity analysis to determine the extent to which this group contributed to dissolution risks for heterogamous marriages. We extended the best-fitting multivariable model, estimating risks separately for marriages where both members reported a current religion and those in which at least one member reported no current religion. These estimates were compared with those from the original model where there was no such distinction between couples.

### Religion Switching

Switching from religion of origin to no religion was the only type whose influence on dissolution could be directly investigated as each respondent answered only one of the subsidiary Census religion questions (current or brought up in). Therefore, our composite measure of religious affiliation (community background) may have been subject to misclassification in cases where either partner switched religion around the time of marriage, perhaps in order to assimilate into the partner’s community of origin (Musick and Wilson 1995). For example, a person with a Protestant community background might convert to Catholicism on marriage and so the couple would be misclassified as Catholic–Catholic. As marriage records were not available for this cohort, we conducted an additional analysis to estimate the likely extent of misclassification. We selected a cohort of 5568 NILS members who were single at the 2001 Census and married to another NILS member at the 2011 Census and calculated the proportion that had switched religion into each marriage type (e.g. Catholic–Catholic, Catholic–Protestant) as a proxy for switching rates in the main cohort. The proportion of people in the new marriages cohort (married between 2001 and 2011) that switched religion (community background) on marriage was 6.1 %, the majority of these initially belonging to no religion or groups other than Protestantism or Catholicism. This proportion varied between marriage types. Only 1.4 % of people entering Catholic–Catholic marriages had switched from Protestantism. 2.0 % of those entering Protestant–Protestant marriages had switched from Catholicism. For those entering Catholic–Protestant marriages, 0.8 % had switched from Catholicism and an equal proportion from Protestantism.

## Results

### Characteristics of Heterogamous and Homogamous Marriages

The majority (91.7 %) of married couples were homogamous by religion with only 5.9 % of marriages spanning the Catholic–Protestant boundary. The remaining 2.4 % of marriages (*n* = 938) consisted of an assortment of different union types involving people of other religious affiliations or none (e.g. Protestant–No religion, No religion–No religion). People in Catholic–Protestant marriages were younger on average than those in homogamous marriages (8.0 % of couples aged 16–34 were in heterogamous marriages compared with 2.4 and 3.8 % in the 35–49 and 50–74 age groups, respectively), and a greater proportion was employed (Table 1). The proportion of marriages with children was greatest for homogamous Catholic marriages, followed by heterogamous marriages and then homogamous Protestant marriages. Similar proportions of couples in each union type owned their own home. A smaller proportion of people in heterogamous marriages were of Northern Ireland origin, and a greater proportion had a degree. People in heterogamous marriages were less likely to live in rural areas than homogamous couples. Homogamous Catholic couples were more likely to live in rural areas and less likely to live in urban areas than both other union types (Table 1).

**Table 1:** Baseline characteristics of married people in Northern Ireland at the 2001 Census by union type

	Protestant–Protestant	Catholic–Catholic	Catholic–Protestant	Other
Cohort (individuals)	21,700	14,592	2352	938
Age				
[16,35)	17.92	20.03	27.17	33.58
[35,50)	39.70	46.13	49.02	43.71
[50,75)	42.39	33.83	23.81	22.71
Economic activity				
Employed	68.69	63.69	75.85	73.67
Inactive	31.31	36.31	24.15	26.33
Higher education				
No degree	82.72	82.26	76.28	69.83
Degree	17.28	17.74	23.72	30.17
Country of birth				
Northern Ireland	92.68	90.54	86.69	66.74
Other	7.32	9.46	13.31	33.26
Marital status				
Both partners first marriage	90.59	96.74	83.59	85.71
At least one partner previously married	9.41	3.26	16.41	14.29
Housing tenure				
Owner occupied	90.20	87.92	89.03	91.47
Social rented	6.58	9.14	7.14	4.69
Private rented	3.22	2.93	3.83	3.84
Children aged 5+				
No	61.08	46.88	53.91	58.64
Yes	38.92	53.12	46.09	41.36
Children aged <5				
No	94.48	88.93	91.50	92.11
Yes	5.52	11.07	8.50	7.89
Rurality				
Rural	33.82	39.27	25.26	35.18
Intermediate	30.39	39.62	39.88	17.91
Urban	35.79	21.11	34.86	46.91

Percentage of individuals in each cohort at baseline given for categorical variables

### Compositional and Contextual Factors

During the decade of follow-up, 2488 marriages (12.6 %) were dissolved (Table 2) and several factors were associated with variation among couples in dissolution risk. Partner age was associated with pronounced variation in dissolution risk among couples; in both unadjusted and adjusted models, there was a gradient of decreasing risk with age among couples where both partners were within the same age group (adjusted odds ratios [ORs] of 1.95 and 0.53, respectively, for the 16–34 and 50–74 age groups compared with 35–49 olds; Table 3). Dissolution risks for marriages heterogamous by age were intermediate of those for the corresponding homogamous unions.

**Table 2:** Number of married couples by religious affiliation in Northern Ireland, 2001, and proportion of marriages dissolved during the subsequent decade

	Protestant–Protestant	Catholic–Catholic	Catholic–Protestant	Other
Couples	10,850	7296	1176	469
Both partners reporting current religion (%)	88.0	95.5	49.7	11.5
Dissolved (%)	11.4	13.0	18.4	18.6

Proportion of couples in which both partners reported current religion (as opposed to religion brought up in)

**Table 3:** Effect of compositional and contextual factors on risk of marital dissolution in Northern Ireland, 2001–2011

Union type	Unadjusted		Adjusted^a^	
	OR	95 %CI	OR	95 % CI
Marital status				
Both partners first marriage	1.00			
At least one partner previously married	1.69	(1.39, 1.84)	1.54	(1.33, 1.79)
Age				
[16,35)–[16,35)	1.65	(1.47, 1.84)	1.95	(1.72, 2.21)
[16,35)–[35,50)	1.50	(1.30, 1.74)	1.53	(1.32, 1.77)
[16,35)–[50,75)	1.03	(0.30, 3.51)	0.69	(0.20, 2.37)
[35,50)–[35,50)	1.00		1.00	
[35,50)–[50,75)	0.82	(0.69, 0.97)	0.78	(0.65, 0.93)
[50,75)–[50,75)	0.50	(0.45, 0.56)	0.53	(0.46, 0.61)
Economic activity				
Employed–employed	1.00		1.00	
Employed–inactive	0.98	(0.89, 1.08)	1.04	(0.94, 1.16)
Inactive–inactive	1.05	(0.94, 1.18)	1.44	(1.25, 1.65)
Higher education				
No degree–no degree	1.00		1.00	
Degree–degree	0.68	(0.58, 0.80)	0.61	(0.52, 0.73)
Degree–no degree	0.82	(0.73, 0.93)	0.80	(0.71, 0.90)
Country of birth				
NI–NI	1.00		1.00	
NI–other	1.18	(1.05, 1.33)	1.13	(1.00, 1.27)
Other–other	1.48	(1.13, 1.93)	1.33	(1.00, 1.77)
Children				
None	1.00		1.00	
Children aged 5+	1.48	(1.36, 1.61)	1.31	(1.18, 1.46)
Children aged <5	1.56	(1.35, 1.79)	1.00	(0.86, 1.16)
Housing tenure				
Owner occupied	1.00		1.00	
Social rented	1.84	(1.61, 2.11)	1.45	(1.25, 1.69)
Private rented	1.88	(1.54, 2.31)	1.51	(1.22, 1.86)
Rurality				
Rural	1.00		1.00	
Intermediate	1.24	(1.11, 1.38)	1.18	(1.06, 1.31)
Urban	1.23	(1.10, 1.37)	1.27	(1.13, 1.41)

Estimated dissolution risk by religion for adjusted model (M4) given in Table 4

*NI* Northern Ireland

^a^Model adjusted for religion, age, economic activity, marital status, education, housing tenure, country of birth, presence of dependent children and rurality

Economic inactivity was associated with increased dissolution risk only following adjustment for other factors. Higher education was associated with decreased dissolution risk in couples homogamous by education (Degree–Degree: OR = 0.61) and to a lesser extent in couples heterogamous by education (OR = 0.80). Couples in which at least one partner was remarried were at a 54 % increased risk of dissolution compared with first marriages. Unadjusted models indicated that people born outside Northern Ireland were at increased risk of marital dissolution but these differences were attenuated following adjustment, indicating that observed differences are largely attributable to the other factors included within the model.

Couples living in rented accommodation were at increased risk of dissolution relative to home owners (ORs = 1.51 and 1.45 for private and social tenants, respectively), but other contextual factors were associated with much less variation in dissolution risk. The presence of young children (under 5 years) in the home was not associated with any change in dissolution risk, but the presence of older children was associated with elevated risk in comparison with couples with no dependent children (OR = 1.31 [1.18, 1.46]). There was a subtle gradient of increasing risk associated with urban residence.

### Religion

Variation in dissolution risk by religion was of similar magnitude to that associated with housing tenure in adjusted models (ORs range: = 1.00, 1.47; Table 4). In unadjusted and age-adjusted models, dissolution risk was slightly higher for homogamous Catholic couples compared with homogamous Protestant couples but this differential was not statistically significant following adjustment for other compositional and contextual factors, indicating that some of the variation among religious groups could be attributed to these factors. As expected Catholic–Protestant marriages remained at substantially increased risk of dissolution relative to homogamous marriages in adjusted models (OR = 1.47 [1.25, 1.73]). Marriages involving those of other religious affiliations were at similarly increased risk.

**Table 4:** Estimated relative risk of dissolution for each union type (ORs and 95 % CIs) in unadjusted and adjusted models

Model	Description	Protestant–Protestant	Catholic–Catholic	Catholic–Protestant	Other	AIC
M1	Unadjusted	1.00	1.16 (1.06, 1.28)	1.75 (1.49, 2.06)	1.78 (1.40, 2.26)	14,914
M2	+Age	1.00	1.10 (1.00, 1.21)	1.50 (1.28, 1.77)	1.46 (1.15, 1.87)	14,555
M3	+Age + employment + education + tenure	1.00	1.06 (0.96, 1.16)	1.54 (1.31, 1.81)	1.54 (1.20, 1.97)	14,394
M4	Fully adjusted^a^	1.00	1.09 (0.99, 1.20)	1.47 (1.25, 1.73)	1.37 (1.06, 1.77)	14,323

^a^Adjusted for age, economic activity, marital status, education, housing tenure, country of birth, presence of dependent children and rurality

### Residential Concentration

The majority of couples lived in areas highly concentrated by community background (60 % in areas with more than 80 % belonging to the majority group). Catholic–Protestant marriages were rare, ranging from just 2.7 % of marriages in some of the most concentrated areas (more than 90 % Catholic, Figure S1) to 8.9 % of marriages in areas where the population proportions of the two communities were more similar (20–30 % of the population Catholic).

We found no evidence for an increase in dissolution risk among Catholic–Protestant couples with degree of residential concentration by religion (Table 5). Inclusion of parameters defining linear, quadratic or cubic relationships between the variables did not significantly improve the fit of the fully adjusted model (i.e. these associations were not statistically significant). Similarly, there was no evidence for associations between residential concentration by religion and dissolution risk for homogamous Protestant and Catholic marriages or other unions. A model including interactions between religious composition and union type was a worse fit than one without interactions (AICs of 14,392 and 14,325, respectively).

**Table 5:** Comparison of multiple linear regression models of marital dissolution risk of couples in Northern Ireland, 2001–2011

Model	Description	AIC	d*f*	LRT	
				*Χ* ^2^	*P*
M4	Base	14,323	23		
M5	+Linear	14,325	24	0.095	0.758
M6	+Linear + quadratic	14,324	25	2.319	0.128
M7	+Linear + quadratic +cubic	14,326	26	0.434	0.519

Base model (M4) is adjusted for age, economic activity, marital status (first or subsequent marriage), education, country of birth, presence of young (<5 years) and older dependent children, housing tenure, settlement type (urban/rural/intermediate) and area of residence (Super Output Area). Subsequent models include polynomial terms related to the religious composition of the area of residence (proportion Catholic) of increasing degree. Model fit assessed using Akaike’s Information Criterion and likelihood ratio tests in comparison with base model

### Sensitivity Analysis

The contribution of marriages in which one or both partners reported no current religion to overall risk estimates was minor. Estimated dissolution risks for couples in which both members declared a current religion from the extended model were very similar to the overall estimates from the best-fitting model using community background (Tables 4 and S2). The 95 % CIs for Catholic–Catholic, Catholic–Protestant and other marriages overlapped in models *M*4 and *M*8. This was to be expected for homogamous marriages where current religion was reported for both members of over 90 % of couples. In only half of Catholic–Protestant marriages did both members report current religion (Table 2), yet estimated dissolution risk was identical in both models. There was a 47 % increase in risk relative to Protestant–Protestant marriages although confidence intervals were wider in the extended model. Catholic–Catholic marriages were also at a 13 % greater risk of dissolution than Protestant–Protestant marriages when both partners reported a current religion. Protestant–Protestant couples in which one or both partners reported no current religion (i.e. where religion brought up in was reported instead) were at 39 % higher risk of dissolution than those in which both partners reported a current religion. Although elevations in risk were observed for Catholic–Catholic and Catholic–Protestant marriages not reporting current religion, these differences were not statistically significant.

## Discussion and Conclusion

Using a cohort of almost 20,000 married couples followed up over a 10-year period, we investigated the roles of heterogamy and residential concentration by religion among a suite of factors that influence risk of marital dissolution. Overall dissolution risks were similar to those observed in a similar study of medium-term (10 years) risk in Great Britain (Feng et al. 2012). We found increased risk associated with youth, indicators of lower socio-economic status (economic inactivity, lower educational attainment and rented accommodation) and a history of previous marriage. These findings are consistent with studies of marriage and partnership dissolution risk in several European countries (Lyngstad and Jalovaara 2010; Jalovaara 2013; Hansen 2005), although previous marriage was associated with a much smaller increase in risk than in a similar study of marital dissolution in Great Britain (Feng et al. 2012). Contrary to some previous work (Bumpass and Sweet 1972; Tzeng 1992), couples heterogamous by age and socio-economic status were not at elevated risk relative to homogamous couples, although the broad classifications for age, economic activity and education that we used may have masked heterogamy effects among particular subgroups. However, these findings may also reflect a greater likelihood of couples heterogamous by socio-economic status dissolving prior to marriage or remaining in an unmarried state (Saarela and Finnäs 2014a). Contextual factors played a more subtle role. Couples living in urban areas had slightly increased risk of marital dissolution relative to those in rural areas, a result also seen in a large Austrian study, where these patterns have been attributed mainly to the increased opportunities to leave an unhappy marriage which the higher incomes associated with urban living provide, along with a lesser influence of conservative rural values (Boyle et al. 2008; Kulu 2012).

In line with expectations, Catholic–Protestant marriages were at substantially greater dissolution risk than either of the corresponding homogamous marriage types. This strong heterogamy effect was of similar magnitude to that observed for Catholic–Protestant marriages in the Netherlands (Kalmijn et al. 2005) but larger than that observed for ethno-linguistically heterogamous couples in Finland (Saarela and Finnäs 2014b). The decreasing magnitude of heterogamy effects in these three contexts indicates that intergroup conflict either in the recent (Northern Ireland) or more distant (Netherlands) past has a negative and persistent influence on marital outcomes exceeding that of ethnic differences alone. Catholic–Protestant marriages remain rare in both the Republic of Ireland (O’Leary 2001) and Northern Ireland (constituting just 5.9 % of couples in our sample) indicating that partner choice is constrained by community boundaries. Together these findings highlight the profound marital divide that remains between the two communities.

Although we found strong evidence associating some contextual factors (housing tenure, urban/rural residence) with dissolution risk, we found no evidence that residential concentration by religion influenced risk of marital dissolution for Catholic–Protestant couples as well as for the other marriage types. The simplest explanation is that in terms of marital stability, the primary influence of religious heterogamy is on the attitudes and expectations of the partners rather than the wider community but this is surprising given that residential segregation along Catholic–Protestant lines is a primary feature of the Northern Ireland sociological landscape. It is also possible that area of residence may not represent the social environment in which couples spend the majority of their time and that concentration by religion in workplaces would be a more accurate measure. However, it was not possible to generate such a measure using this dataset. Finally, internal migration may also have obscured potential relationships between concentration by religion and dissolution risk with Catholic–Protestant couples avoiding community disapproval by migrating to areas less concentrated by religion. We found some indication that whilst remaining a small minority in all areas, Catholic–Protestant couples were more common in areas less concentrated by religion and surveys of social attitudes have revealed that heterogamous couples have a greater preference for mixed religion neighbourhoods than homogamous couples (Lloyd and Robinson 2011). This hypothesis could be investigated further if marriage records were linked to the NILS, allowing the number and timing of moves in relation to date of marriage to be quantified. It might also indicate whether increased dissolution risk for other types of heterogamous couples is partially due to the detrimental effects of repeated moving on marital stability (Boyle et al. 2008).

We investigated the extent to which a possible selection effect influenced dissolution risk for heterogamous couples; that is, that those prepared to marry outside their religion of origin had less traditional attitudes to marriage and hence were more likely to experience dissolution. As the Census does not elicit information on religious practice, failure to declare a current religious affiliation (to any group) was used as a measure of detachment from religion brought up in (e.g. switch from Catholic to No religion). Whilst there was some evidence that lack of current affiliation was associated with increased dissolution risk for homogamous couples and that in half of the heterogamous couples at least one member reported no current affiliation, we found no difference in risk for heterogamous couples regardless of current affiliation. These findings suggest that renouncing religion of origin for a neutral position is insufficient to mitigate the tensions caused by marriage across the Catholic–Protestant divide.

However, we were unable to determine whether heterogamous couples in which either or both members had completely switched religion (e.g. Catholic to Protestant) were at elevated dissolution risk as only one response, either current religion or religion brought up in was recorded in the Census. Inclusion of marriage records within the NILS would allow assessment of dissolution risk for couples in which either partner completely switched religious affiliation either prior to or during marriage to investigate further whether this strategy reduces risk for couples with different religious backgrounds. If switching couples remained at higher dissolution risk than truly homogamous couples (as suggested by the sensitivity analysis), then our risk estimates for the latter may have been inflated. However, our analysis of switching in the younger ‘new marriages’ cohort indicates that switching between Catholicism and Protestantism at entry into homogamous marriages remains rare in Northern Ireland (<2 % of people) and so any bias due to switching is likely to have been small.

Incorporation of marriage records would also remove a limitation common to this and similar studies (Feng et al. 2012; Hansen 2005), in that we were unable to measure the duration of marriages in our dataset. Dissolution risk varies with time since marriage, following a characteristic humped trajectory (Kulu 2014) and has also increased with subsequent birth cohorts in the UK (Chan and Halpin 2003). Adjustment for age at study entry is unlikely to account for these effects completely and so the precision of our estimates of dissolution risk and heterogamy effects would be improved with the inclusion of duration data, which would also allow adjustment for age at marriage, an established risk factor for dissolution.

Building on our finding that Catholics and Protestants in Northern Ireland were segregated by marriage as well as area of residence at the 2001 Census, an interesting line of future work would be to investigate whether this boundary exerts a similar influence over less formal relationships, specifically between cohabiting couples. There is evidence that cohabitation is relatively common among couples heterogamous by religion in Northern Ireland compared with homogamous couples (Lloyd and Robinson 2011; McAloney 2013). Given that cohabiting couples are more likely to separate than married couple (Lyngstad and Jalovaara 2010; Jalovaara 2013), an extension to this study might involve construction of a composite measure of dissolution risk for heterogamous couples based on outcomes of both marriages and cohabiting relationships. Such estimates could be compared with those observed in Finland for couples heterogamous by language, a context in which the two ethno-linguistic groups have no recent history of conflict (Saarela and Finnäs 2014b). Finally, it would be interesting to investigate whether the Northern Ireland population has followed the trend towards increased secularism and intermarriage among religious groups observed in other Western countries (Frimmel et al. 2012; Kalmijn 1991; Lehrer 1998; Raab and Holligan 2012; Rosenfeld 2008) as peaceful interaction between the two communities becomes the norm. The younger age of heterogamous couples in our dataset indicates that this may be the case.

Using a large linked dataset, we have shown that the few marriages crossing the Catholic–Protestant divide in Northern Ireland are at substantially increased risk of dissolution in comparison with homogamous marriages. Contrary to expectation, there was no association between residential concentration by religion and dissolution risk for Catholic–Protestant marriages, indicating that attitudinal differences between partners may have a greater influence on existing marriages than the wider community. There are signs that Catholic–Protestant relationships are more common among the young, boding well for future prospects of community integration through intermarriage in post-conflict Northern Ireland.

## Electronic supplementary material

Below is the link to the electronic supplementary material.

Supplementary material 1 (DOCX 1767 kb)
