# Supplementary material for: Influence of Heterogamy by Religion on Risk of Marital Dissolution: A Cohort Study of 20,000 Couples
Source: Eur J Popul. 2016 Sep 19;33(1):87–107. doi: 10.1007/s10680-016-9398-9 (PMC5318477; doi:10.1007/s10680-016-9398-9)
Supplement: Supplementary file 1 — Supplementary material 1 (DOCX 1767 kb) [file 10680_2016_9398_MOESM1_ESM.docx]

# Supplementary Information

Figure S1. Proportion of marriages in Census Super Output Areas (SOAs) by type and proportion of the SOA population Catholic.

**
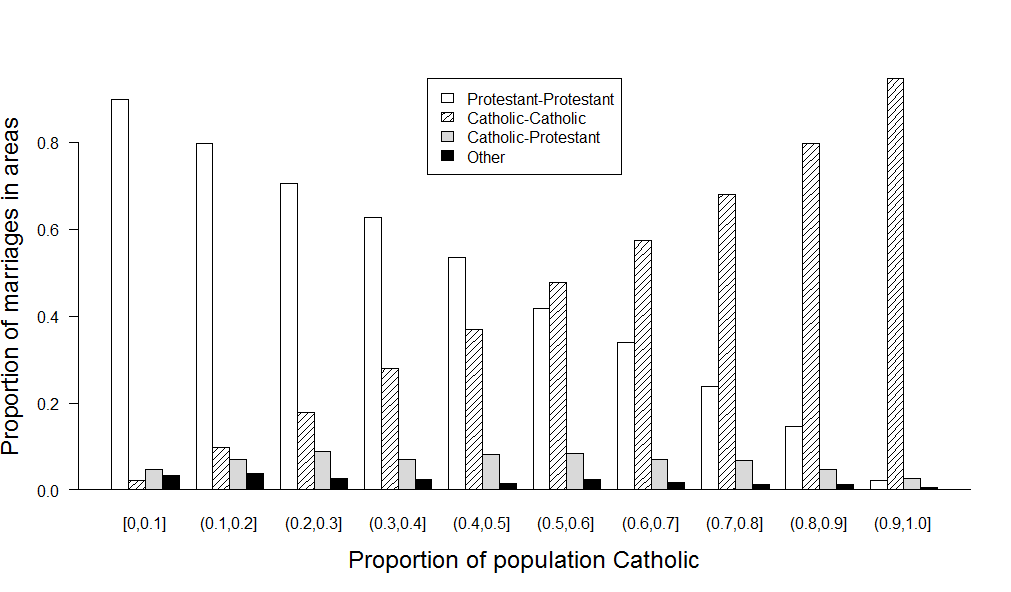
**

Table S1. Matching rates across Censuses (2001-2011) of married couples in Northern Ireland by religious affiliation.

|  | Protestant-Protestant | Catholic-Catholic | Catholic-Protestant | Other |
| --- | --- | --- | --- | --- |
| Couples 2001 | 12,569 | 8,444 | 1315 | 572 |
| Deaths | 1485 | 929 | 77 | 44 |
| Neither partner found^1^ | 234 | 219 | 62 | 59 |
| One partner found | 546 | 521 | 87 | 31 |
| Both partners found | 10,304 | 6,775 | 1089 | 438 |
| Couples followed up | 10,850 | 7296 | 1176 | 469 |
| Overall matching rate (%) | 86.3 | 86.4 | 89.4 | 82.0 |
| Overall matching rate excluding deaths (%) | 97.9 | 97.1 | 95.0 | 88.9 |

^1^Or records removed to comply with NILS disclosure rules.

Table S2. Estimated relative risk of marital dissolution for couples in Northern Ireland, 2001-2011 by union type (ORs and 95% CIs).

| Current religion or religion brought up in | Protestant-Protestant | Catholic-Catholic | Catholic-Protestant | Other |
| --- | --- | --- | --- | --- |
| Both partners reported current religion. | 1.00 | 1.13 (1.02, 1.25) | 1.47 (1.17, 1.84) | 2.45 (1.29, 4.65) |
| One or both partners reported no current religion. | 1.39 (1.17, 1.64) | 1.33 (0.98, 1.82) | 1.63 (1.41, 2.04) | 1.35 (1.03, 1.78) |

Where a partner reported no current religion, couple type was assigned using the religion brought up in. Model (M8) adjusted for age, economic activity, marital status, education, housing tenure, country of birth, presence of dependent children and rurality.
